# Effects of walking training with blood flow restriction on the hemodynamics and perceptual responses among sedentary college students: A randomized crossover trial

**DOI:** 10.1371/journal.pone.0352582

**Published:** 2026-07-13

**Authors:** Yuke Zhu, Ying Wang, Siyu Yu, Dejiang Sun, Xiuyi Ji, Wenbin Yuan, Min Ma, Huagang Hu

**Affiliations:** School of Nursing, Suzhou Medical College of Soochow University, Suzhou, Jiangsu Province, China; Universiti Malaya, MALAYSIA

## Abstract

College students often present a sedentary lifestyle. Low-intensity walking training with blood flow restriction (WT-BFR) may offer health benefits comparable to moderate-to-high intensity walking training without restriction, yet its effects in sedentary college students remain unclear. This study aimed to examine the effects of WT-BFR at different limb occlusion pressure (LOP) on hemodynamic and perceptual responses in sedentary college students using a randomized crossover design. The study was registered on the China Clinical Trial Registry (ChiCTR2500097728 25/02/2025). A total of 60 participants completed the 5-minute WT-BFR with varying LOPs (i.e., 0%, 40%, 60%, and 80%). Hemodynamic parameters (blood pressure and heart rate) were measured before, immediately after, and 5 minutes post-intervention. Meanwhile, perceptual responses (perceived exertion and discomfort) and step numbers were recorded post-intervention. For the hemodynamic parameters, only 60% LOP showed a larger increase in heart rate after training than 0% LOP (walk training without BFR) condition (3.82, 95%CI: 0.72 to 6.91, p = 0.016, beats/min), representing a relative increase of approximately 5.6% from baseline. With the increase of LOP, perceived exertion and discomfort were increased significantly (p < 0.05), and the step numbers were reduced (p < 0.05). Based on participant perception, an LOP range of 40%–60% is recommended. Clinically, the observed cardiovascular changes were modest and within safe ranges for healthy young adults. The long-term health effects of low-intensity WT-BFR among college students warrant further investigation.

## Introduction

College students exhibit a notably low level of physical activity and represent a typical sedentary population [1]. Studies have revealed that the average daily sedentary time among college students reaches 9.83 hours [2]. This sedentary lifestyle poses substantial health risks, including overweight/obesity [3], cancer [4], type 2 diabetes [5], cardiovascular disease mortality [5], and all-cause mortality [6]. Regular physical exercise has been demonstrated to substantially enhance both physiological and psychological well-being among college students, including improvements in cardiorespiratory fitness [7], sleep quality [8], and the alleviation of anxiety and depression [9]. However, the proportion of students engaging in regular exercise remains limited [1]. This phenomenon may be attributable to multiple exercise barriers, particularly academic-related factors such as time constraints and demanding coursework. In addition, subjective factors, including lack of personal exercise habits, limited interest, discomfort associated with high-intensity exercise, and insufficient sports skills, collectively contribute to reduced exercise participation [10].

The World Health Organization (WHO) recommends that adults aged 18–64 complete at least 150 minutes of moderate-intensity aerobic activity, 75 minutes of vigorous-intensity aerobic activity, or some combination of both, weekly, as well as muscle strengthening activities involving major muscle groups on two or more days per week [11]. However, the combination of limited time and various subjective factors collectively hinders college students from meeting the WHO’s minimum threshold for health-promoting physical activity. As a fundamental form of physical activity, walking represents a substantial component of daily movement in this population. Nevertheless, it is typically performed at low intensity—a level that falls below the minimum recommended by the WHO.

Blood flow restriction (BFR) training has emerged as a scientifically reliable exercise modality, characterized by the application of specialized compression devices (e.g., pneumatic cuffs, elastic bands) to proximal limb segments, thereby reducing arterial inflow and blocking venous outflow to enhance training stimulus [12]. Walking training with BFR (WT-BFR) represents an innovative exercise paradigm that combines low-intensity dynamic training with controlled vascular occlusion. This time-efficient training method, characterized by its low intensity and short-duration parameters, has been demonstrated to be effective across diverse populations [13]. Typically, low-intensity exercise combined with BFR demonstrates superior effects compared to equivalent exercise without BFR, while producing outcomes comparable to high-intensity exercise without BFR. In overweight middle-aged males, WT-BFR has been shown to significantly improve systemic inflammatory markers, optimize lipid profiles, and modulate hematological indices [14]. For elderly populations, particularly those with knee osteoarthritis, WT-BFR not only exhibits excellent feasibility but also effectively enhances physical function [15,16]. These findings underscore the significant application value of WT-BFR as a safe and effective exercise intervention for improving health outcomes in difference of populations.

Research indicates that the duration of a single BFR training session typically ranges from 5 to 20 minutes, with the BFR applied to both small and large muscle groups (e.g., upper and lower limbs, either unilaterally or bilateral [17,18]. The pressure for BFR is generally set between 40% and 80% of the limb occlusion pressure (LOP), where LOP is defined as the minimum pressure required to completely occlude blood flow in the limb. The exercise modalities primarily include aerobic exercise (e.g., cycling and walking) and resistance exercise (e.g., deep squat and knee extension) [19–21]. Existing studies suggest that higher BFR pressures may enhance cardiovascular responses but might also induce significant discomfort [22,23]. Therefore, prior to implementing BFR training among sedentary college students, it is necessary to investigate the effects of different LOP levels on hemodynamic responses in this population.

WT-BFR may offer college students a low-intensity, time-efficient, and highly effective exercise modality. However, researches about WT-BFR on sedentary college students remains unreported. This study aims to investigate the effects of different intensities of BFR on hemodynamic and perception responses in sedentary college students and to evaluate the acceptability of various WT-BFR intensities in this population. The findings will help establish the safety profile of WT-BFR for sedentary college students and lay the groundwork for further research into the potential benefits of BFR exercise.

## Materials and methods

### Study design

**Fig 1:**
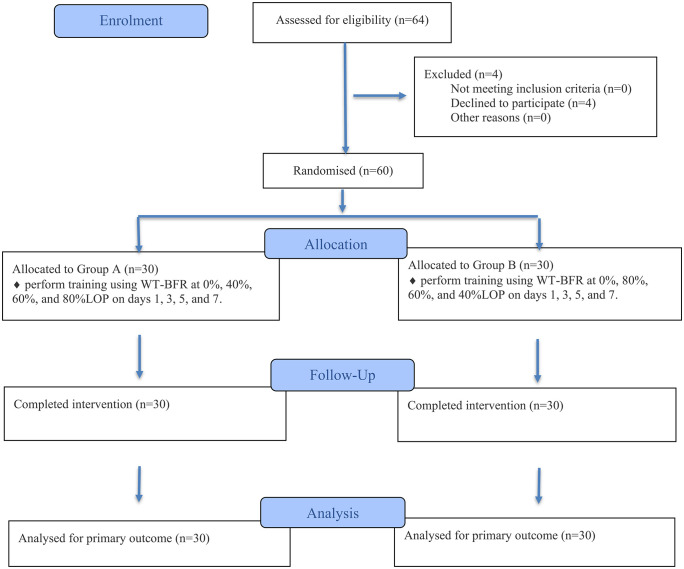
CONSORT Flow diagram. WT-BFR, walking training with blood flow restriction.

This is a randomized crossover study. The trial protocol was approved by the Soochow University Ethics Committee (Approval No.: SUDA20241006H03) and conducted in accordance with the Declaration of Helsinki (2013). This study received ethical approval prior to the initiation of the research in October 2024. This trial has been retrospectively registered with the registry (Registration number: ChiCTR2500097728 25/02/2025) to enhance research transparency and ensure adherence to best practices. We confirm that all related and ongoing trials are now registered. No modifications to the study design, outcome measures, or analytical plan were made after study commencement. Before the study, written informed consent was obtained from each participant (**Fig 1**).

### Setting and participants

Participants were recruited at Soochow University from October 7th, 2024 to December 7th, 2024. Eligibility criteria included: (ⅰ) undergraduate or postgraduate students aged 18 or above; (ⅱ) low physical activity (<1500 MET-min/week assessed by International Physical Activity Questionnaire, IPAQ) [24]; (ⅲ) prolonged sedentary time (≥ 6 hours/day); (ⅳ) frequency exercise (<3 weekly sessions of <30 minutes duration); and (ⅴ) absence of exercise contraindications (e.g., severe cardiovascular diseases, respiratory diseases, musculoskeletal disorders, and metabolic diseases). Exclusion criteria were: (i) engaged in regular physical activity or exercise (>3 times/week of moderate to high-intensity exercise, each session lasting at least 20 minutes); (ⅱ) alcohol, nicotine, drug, and medications use; and (ⅲ) pregnant.

### Sample size calculation

The primary outcome of this study was systolic blood pressure (SBP). The sample size was calculated using a paired design-mean comparison algorithm $\text{n}=\frac{{\left({\text{Z}}_{\alpha }+{\text{Z}}_{\beta }\right)}^{\text{2}}*\sigma^{\text{2}}}{\delta^{\text{2}}}$[25]. According to published research, the standard deviation of systolic blood pressure (SBP) among college students was 15 mmHg [26]. Based on the reference literature, a difference in SBP of less than 10 mmHg between two different exercises was considered insignificant. Hence, the mean difference between the two groups was set at 10 mmHg. Type I error α was set at 0.05. Therefore, Z_0.05_ = 1.96. Type II error β was set at 0.1, Z_β_ = 1.28. The calculated sample size was 24 participants per group. Considering a 20% dropout rate, we planned to recruit 60 subjects.

### Intervention protocol

**Fig 2:**
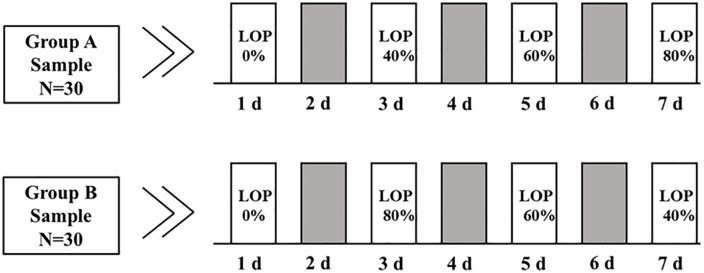
Study Design. The timing of exercise sessions is shown. LOP, limb occlusion pressure; shaded blocks, rest day.

This study employed a randomized crossover design to control for potential order effects related to varying LOP sequences. The two sequences (0%−40%−60%−80% LOP and 0%−80%−60%−40% LOP) were designed as reverse orders to balance the presentation of each pressure level across different time points, thereby minimizing the influence of order and carryover effects. An independent researcher was responsible for grouping allocation using an online simple randomization website (www.sealedenvelope.com). Participants in group A performed training using WT-BFR at 0% (i.e., walking training without BFR), 40%, 60%, and 80%LOP on days 1, 3, 5, and 7 of the trial, respectively. Participants in Group B performed training using WT-BFR at 0%, 80%, 60%, and 40%LOP on days 1, 3, 5, and 7 of the trial, respectively (**Fig 2**). Each training session was followed by a one-day interval before the next trial. According to data from our pilot study, the 1-day washout period implemented in this study was adequate for maintaining the reliability of outcomes.

**Fig 3:**
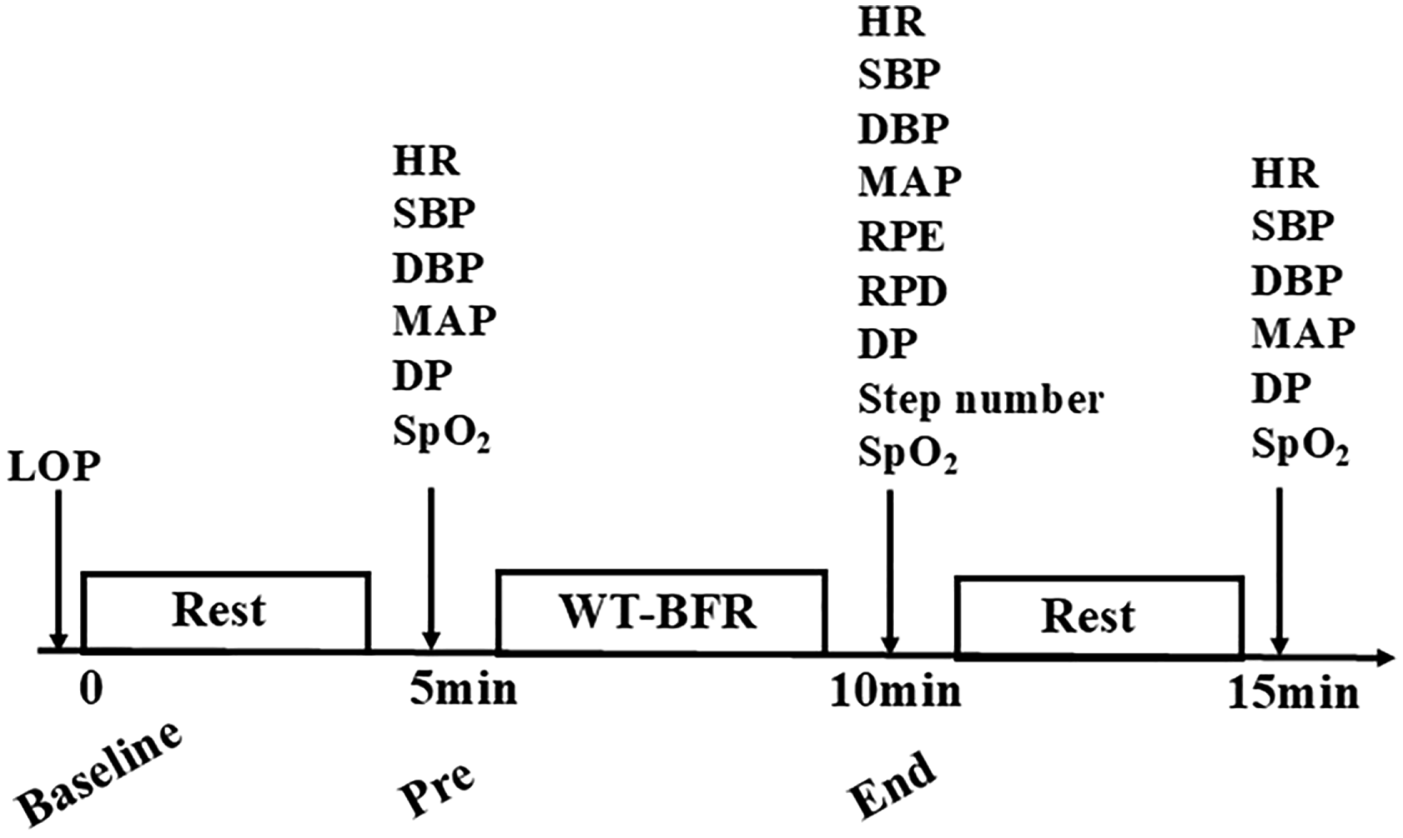
Single session timeline. The timing of measures is indicated on the single session timeline. LOP, limb occlusion pressure; HR, heart rate; SBP, systolic blood pressure; DBP, diastolic blood pressure; MAP, mean arterial pressure; RPE, rating of perceived exertion; RPD, rating of perceived discomfort; SpO_2_, pulse oxygen saturation; WT-BFR, walking training with blood flow restriction. DP, double product.

The experimental procedure of each training session consisted of three stages: (i) 5 minutes rest before training; (ii) 5-minute training; and (iii) 5-minute rest (**Fig 3**). The experiment was conducted on a horizontal outdoor playground within Soochow University between 7:00 and 20:00 on each experimental day (days 1, 3, 5, 7) by two researchers. Researchers have undergone unified and professional training. The WT-BFR was performed at a moderate walking speed (50–83 meters/minute). Each participant determined their walking speed based on their daily walking speed, which remained constant throughout the training. It ensured that different WT-BFR exercise intensity did not exceed the habitual walking exercise performed by the participants.

For BFR, the restriction was applied during each exercise bout only, using a uniform device: A&B Physiotherapy LTD, Theratools, China. The device was a 10 cm wide inflatable cuff with an external, removable, pressure-displaying, and manually inflatable deflator. Measurement of LOP was completed before the first BFR exercise session. An inflatable cuff was placed on the participant’s upper thigh (near the groin) with an elasticity to accommodate two fingers in the uninflated state. A portable ultrasound monitor (Edan SD 3 Vascular Ultrasound Pocket Doppler, Edan USA) was used to be placed on the ipsilateral intra-ankle anterior tibial artery course. When arterial pulsation was monitored, the inflatable cuff was pressurized until the monitored arterial pulsation disappeared, at which time the intracuff pressure was the LOP of the limb on that side. This approach has demonstrated excellent reliability, with intra- and inter-rater ICC values of 0.99 and 0.98, respectively [27]. The LOP was individually measured and set for each lower extremity (i.e., right and left) during all training sessions. For the safety consideration, when the LOP was measured above 300 mmHg, the LOP was recorded as 300 mmHg [28].

### Outcomes measurements

The primary outcome was SBP and second outcomes include hemodynamic indicators, perceived response, step number, and adverse events during training sessions. For all sessions, hemodynamic measures were taken immediately before, immediately after each exercise bout, and 5 minutes after rest. Hemodynamic measures included heart rate, SBP, diastolic blood pressure (DBP), mean arterial pressure (MAP), double product, and pulse oxygen saturation (SpO_2_). Heart rate, SBP, and DBP were measured using a smart blood pressure monitor (Yuwell, YE660AR, Danyang, China). Blood pressure measurements were obtained with participants in a seated position. To ensure consistency, all measurements were performed by one researcher. Due to the nature of the intervention, the researcher performing the measurements was not blinded to group allocation. Double product was calculated using the formula DP=SBP×heart rate, MAP was calculated using the formula $\text{MAP}=\frac{\text{SBP}+\text{2}\times \text{DBP}}{\text{3}}$[29], and SpO_2_ was measured using a medical pulse oximeter (Yuwell, YX306, Danyang, China).

Perceptual response including rating of perceived exertion (RPE), rating of perceived discomfort (RPD). Immediately after each exercise bout, participants were asked to provide RPE on a Borg scale ranging from 6 (no exertion) to 20 (maximal exertion) and RPD using a modified Borg scale ranging from 0 (no discomfort) to 10 (maximal discomfort). Step number was recorded during each training sessions using electronic pedometer (Polygon, ABL-208, Shenzhen, China).

Any physical discomfort, such as chest pain, chest tightness, dyspnea, pain in the lower limbs, symptomatic high or low blood pressure, and mental discomfort was recorded and reported back to the research team and the doctor for timely management. At the end of the BFR exercise, the researcher checked the condition of the skin at and below the cuff coverage area, such as bruising or edema.

### Statistical analysis

The database was established using Epidata 3.1. The statistical analyses were performed using SPSS 25.0 (IBM, Chicago, IL). Normally distributed measurement data were described using mean with standard deviation (SD), while non-normally distributed data were described using median and interquartile range [M (P_25_, P_75_)]. Categorical data were described using frequency (percentage). For the primary outcome (SBP), a between-group difference of 10 mmHg was considered the minimal clinically important difference based on prior literature [26]. One-way ANOVA was used to compare the RPE, RPD, and step number among different percentage LOP conditions. Generalized estimating equation (GEE) model was used to analysis the effects of different levels of LOP on hemodynamics in sedentary college students. The effect was expressed as mean difference with 95% confidence intervals (95%CI). Two-side statistically significant level was set at 0.05. No adjustment was made for the multiple statistical tests conducted. In addition, there were no missing data.

## Results

Sixty college students, 50% male (n = 30), aged 20.9 ± 3.6 years old, were included in this study (**Table 1**). Adherence to the training protocol was 100%. No adverse signs or symptoms were reported during all training sessions. Hemodynamic indicators measured at various time points under different LOP levels were presented in Table 2.

**Table 1:** Baseline characteristics of participants.

Variables	Total (n = 60)	Range (95% CI)
Age, year	20.9 ± 3.6	18–27 (20.0, 21.9)
Sex		
Male	30 (50)	–
Female	30 (50)	–
Height, cm	169.7 ± 7.6	155–185 (167.7, 171.7)
Dry weight, kg	64.4 ± 13.8	42–120 (60.8, 68.0)
Body mass index, kg/m^2^	22.3 ± 3.9	17.0–39.2 (21.3, 23.3)
Thigh circumference, cm		
Left	54.9 ± 5.8	40.0–70.0 (53.4, 56.4)
Right	55.0 ± 5.7	41.0–70.0 (53.5, 56.4)
Limb occlusion pressure, mmHg		
Left	194.1 ± 32.0	140–290 (185.0, 201.6)
Right	195.0 ± 31.0	150–282 (185.8, 202.1)

Notes: data are givens as number (percentage) or mean ± standard deviation (95%CI).

Abbreviations: 95% CI, 95% confidence interval.

**Table 2:** Hemodynamic parameter in different conditions.

Outcomes	0%LOP (95% CI)	40%LOP (95% CI)	60%LOP (95% CI)	80%LOP (95% CI)
Baseline				
Outcomes	0%LOP	40%LOP	60%LOP	80%LOP
SBP, mmHg	119.98 ± 14.56 (116.22, 123.74)	117.90 ± 13.45 (114.42, 121.38)	117.28 ± 13.45 (113.81, 120.76)	116.80 ± 13.82 (113.23, 120.37)
DBP, mmHg	75.38 ± 11.33 (72.46, 78.31)	75.98 ± 12.05 (72.87, 79.10)	73.12 ± 10.23 (70.47, 75.76)	73.90 ± 9.25 (71.51, 76.29)
MAP, mmHg	90.25 ± 11.02 (87.40, 93.10)	89.96 ± 11.21 (87.06, 92.85)	87.84 ± 10.00 (85.25, 90.42)	88.20 ± 9.77 (85.68, 90.72)
HR, beats/min	78.23 ± 11.63 (75.23, 81.24)	77.97 ± 13.34 (74.52, 81.41)	75.73 ± 10.87 (72.93, 78.54)	75.82 ± 12.60 (72.56, 79.07)
DP, beats/min mmHg	9403.73 ± 1898.52 (8913.29, 9894.17)	9249.13 ± 2188.42 (8683.80, 9814.46)	8925.87 ± 1911.90 (8431.97, 9419.76)	8883.40 ± 2029.66 (8359.08, 9407.72)
SpO_2_, %	98.48 ± 1.20 (98.17, 98.79)	98.45 ± 1.52 (98.06, 98.84)	98.65 ± 1.46 (98.27, 99.03)	98.50 ± 1.30 (98.17, 98.83)
T1				
SBP, mmHg	122.35 ± 14.70 (118.55, 126.15)	119.30 ± 12.50 (116.07, 122.53)	118.23 ± 13.48 (114.75, 121.71)	118.80 ± 13.03 (115.43, 122.17)
DBP, mmHg	76.82 ± 12.55 (73.57, 80.06)	75.38 ± 11.35 (72.45, 78.31)	74.60 ± 9.20 (72.22, 76.98)	75.07 ± 9.52 (72.61, 77.53)
MAP, mmHg	91.99 ± 11.61 (89.00, 95.00)	90.02 ± 10.22 (87.38, 92.66)	89.14 ± 8.89 (86.85 91.44)	89.64 ± 9.37 (87.22, 92.07)
HR, beats/min	79.38 ± 13.88 (75.80, 82.97)	80.12 ± 13.26 (76.69, 83.54)	80.70 ± 12.39 (77.50, 83.90)	79.87 ± 13.19 (76.46, 83.27)
DP, beats/min mmHg	9753.45 ± 2301.29 (9158.96, 10347.94)	9582.50 ± 2011.35 (9062.91, 10102.09)	9604.18 ± 2186.11 (9039.45, 10168.92)	9516.62 ± 2018.98 (8995.06, 10038.17)
SpO_2_, %	98.62 ± 1.17 (98.32, 98.92)	98.67 ± 1.23 (98.35, 98.98)	98.77 ± 1.11 (98.48, 99.05)	98.65 ± 1.46 (98.27, 99.03)
T2				
SBP, mmHg	115.47 ± 12.60 (112.21, 118.72)	115.02 ± 12.93 (111.68, 118.36)	113.33 ± 11.97 (110.24, 116.43)	112.60 ± 12.09 (109.48, 115.72)
DBP, mmHg	73.72 ± 11.04 (70.87, 76.57)	73.50 ± 9.98 (70.92, 76.08)	72.87 ± 9.19 (70.49, 75.24)	74.22 ± 10.41 (71.53, 76.91)
MAP, mmHg	87.63 ± 10.49 (84.92, 90.34)	87.34 ± 10.00 (84.75, 89.92)	86.36 ± 9.32 (83.95, 88.76)	87.01 ± 9.55 (84.55, 84.48)
HR, beats/min	77.87 ± 12.56 (74.62, 81.11)	78.82 ± 12.44 (75.60, 82.03)	78.47 ± 11.48 (75.50, 81.43)	75.92 ± 11.70 (72.89, 78.94)
DP, beats/min mmHg	9010.63 ± 1889.08 (8522.63, 9498.64)	9103.57 ± 1976.33 (8593.03, 9614.11)	8929.63 ± 1851.69 (8451.29, 9407.98)	8551.98 ± 1654.70 (8124.53 8979.44)
SpO_2_, %	98.75 ± 1.07 (98.47, 99.03)	98.65 ± 1.30 (98.31, 98.99)	98.75 ± 1.22 (98.44, 99.06)	98.55 ± 1.17 (98.25, 98.85)

Notes: data are givens as mean ± standard deviation (95%CI). T1, immediately after completing the intervention; T2, 5 minutes after completing the intervention.

Abbreviations: 95% CI, 95% confidence interval; LOP, limb occlusion pressure; SBP, systolic blood pressure; DBP, diastolic blood pressure; MAP, mean arterial pressure; HR, heart rate; DP, double product SpO_2_, pulse oxygen.

A one-way ANOVA revealed statistically significant differences in step number, RPE and RPD among the different conditions (all *p* < 0.05, Table 3). Step number was significantly lower in the 60%LOP (453.67 ± 96.93, *p* < 0.05) and 80%LOP (418.42 ± 126.36, *p* < 0.05) conditions compared to the walking training without BFR (517.82 ± 78.22). Moreover, compared with the 40%LOP condition (477.25 ± 95.04), step number in the 80%LOP condition (418.42 ± 126.36, *p* < 0.05) was also significantly reduced. RPE and RPD scores increased progressively with higher LOP intensity. Compared with walking training without BFR, significant differences were observed in 40%, 60% and 80%LOP conditions (all *p* < 0.05). Furthermore, RPE and RPD scores in the 60%LOP and 80%LOP conditions were all significantly higher than those in the 40%LOP condition (all *p* < 0.05).

**Table 3:** Step number and perceptual responses in different conditions.

	0%LOP (95% CI)	40%LOP (95% CI)	60%LOP (95% CI)	80%LOP (95% CI)
Step number	517.82 ± 78.22 (497.61, 538.02)	477.25 ± 95.04 (452.70, 501.80)	453.67 ± 96.93 ^a^ (428.63, 478.71)	418.42 ± 126.36 ^a,b^ (385.77, 451.06)
RPE	7.42 ± 1.66 (6.99, 7.85)	9.28 ± 2.17 ^a^ (8.72, 9.84)	10.67 ± 2.36 ^a,b^ (10.06, 11.28)	13.10 ± 2.86 ^a,b,c^ (12.36, 13.84)
RPD	0.02 ± 0.13 (−0.02, 0.05)	0.98 ± 1.31 ^a^ (0.65, 1.32)	2.27 ± 1.76 ^a,b^ (1.81, 2.72)	4.55 ± 2.76 ^a,b,c^ (3.84, 5.26)

Notes: data are givens as mean ± standard deviation (95%CI). ^a^ *P* < 0.05 compared with 0%LOP; ^b^ *P* < 0.05 compared with 40%LOP; ^c^ *P* < 0.05 compared with 60%LOP. Abbreviations: LOP, limb occlusion pressure; RPE, rating of perceived exertion; RPD, rating of perceived discomfort.

Abbreviations: 95% CI, 95% confidence interval.

The intervention effect on SBP, DBP, heart rate, SpO_2_, MAP and double product, as analyzed by the GEE model, was shown in Table 4. At T1 and T2, compared with walking training without BFR, none of the BFR conditions (i.e., 40%, 60% or 80% LOP) showed statistically significant changes from baseline in SBP, DBP, SpO₂, MAP, or double product (all *p* > 0.05). However, heart rate increased significantly in the 60% LOP condition at T1 (mean changes and 95% CI: 3.82, 0.72 to 6.91, *p* = 0.016) and T2 (3.10, 0.411 to 5.79, *p* = 0.024) than 0%LOP.

**Table 4:** The intervention effect on hemodynamic parameter by generalized estimating equation model.

Time points	Between-group changes (from baseline) difference at each time point						*P* value for Group × Time interaction effect
	40% LOP vs. 0% LOP		60% LOP vs. 0% LOP		80% LOP vs. 0% LOP		
	Estimate (95% CI)	*P* value	Estimate (95% CI)	*P* value	Estimate (95% CI)	*P* value	
SBP							
T1	−0.97 (−4.40, 2.46)	0.581	−1.42 (−5.12, 2.28)	0.453	−0.37 (−4.36, 3.63)	0.857	
T2	1.63 (−1.47, 4.37)	0.302	0.57 (−2.59, 3.73)	0.725	0.32 (−3.20, 3.83)	0.860	<0.001
DBP							
T1	−2.03 (−5.27, 1.20)	0.218	0.05 (−2.96, 3.06)	0.974	−0.27 (−3.12, 2.59)	0.855	
T2	−0.82 (−3.35, 1.72)	0.528	1.42 (−1.24, 4.08)	0.297	1.98 (−0.76, 4.73)	0.156	0.012
HR							
T1	1.00 (−2.40, 4.40)	0.564	3.82 (0.72, 6.91)	0.016	2.90 (−0.35, 6.15)	0.080	
T2	1.22 (−1.85, 4.29)	0.437	3.10 (0.411, 5.79)	0.024	0.467 (−2.32, 3.25)	0.742	<0.001
SpO_2_							
T1	0.08 (−0.46, 0.63)	0.764	−0.02 (−0.51, 0.47)	0.947	0.02 (−0.56, 0.59)	0.955	
T2	−0.07 (−0.56, 0.42)	0.789	−0.17 (−0.69, 0.35)	0.893	−0.22 (−0.63, 0.20)	0.305	0.755
MAP							
T1	−1.68 (−4.35, 1.00)	0.218	−0.44 (−3.04, 2.16)	0.741	−0.30 (−2.90, 2.30)	0.821	
T2	0.01 (−2.07, 2.07)	1.000	1.13 (−1.04, 3.31)	0.307	1.43 (−0.85, 3.71)	0.219	<0.001
DP							
T1	−16.35 (−519.73, 487.03)	0.949	328.60 (−168.43, 825.63)	0.195	283.50 (−219.05, 786.05)	0.269	
T2	247.53 (−192.72, 687.79)	0.270	396.87 (−27.43, 821.16)	0.067	61.68 (−391.68, 515.05)	0.790	<0.001

Notes: T1, immediately after completing the intervention; T2, 5 minutes after completing the intervention; Numbers in bold represent statistically significant results.

Abbreviations: 95% CI, 95% confidence interval; SBP, systolic blood pressure; DBP, diastolic blood pressure; MAP, mean arterial pressure; HR, heart rate; DP, double product SpO_2_, Pulse oxygen.

## Discussion

### Overall findings

This study investigated the hemodynamic response of walking training combined with varying levels of LOP among sedentary college students. The results indicate that, compared with walking training without BFR, the application of 40%, 60%, and 80% LOP during a 5-minute walking exercise did not induce significant changes in hemodynamic parameters, including SBP, DBP, MAP, double product, and SpO_2_, except for heart rate. These findings support the cardiovascular safety of WT-BFR in sedentary college students.

These findings indicate that WT-BFR demonstrates cardiovascular safety comparable to conventional exercise protocols recommended for sedentary college populations. Under the 60% LOP condition, there was a certain change in heart rate. However, under higher BFR conditions, the difference in heart rate changes did not show statistical significance. The observed increase in heart rate may be partly attributed to enhanced stimulation of group III and IV afferent nerve fibers under BFR, which may augment the exercise pressor reflex [30]. The relative hemodynamic stability observed in sedentary college students during WT-BFR may be partially explained by their healthy cardiovascular reserve, the low-intensity aerobic nature of the exercise, and the use of moderate cuff pressures (40–80% LOP), all of which are within ranges previously suggested not to increase acute hemodynamic risk in populations without cardiovascular comorbidities [19,31,32].

The participants’ perception of WT-BFR will affect their compliance with the intervention plan. Therefore, it is also very important to assess the participants’ perception of WT-BFR among college students. The results showed that perceptual responses were significantly higher in the WT-BFR condition than in the walking training without BFR condition. Specifically, the RPE and RPD demonstrated a marked increase across the 40%, 60%, and 80% LOP conditions accompanied by a significant reduction in step number during the 5-minute walking protocol. While minimal perceptual differences were observed at 40% LOP, a statistically significant increase was evident at 80% LOP. However, although higher LOP levels (e.g., 80%) did not substantially affect hemodynamic responses, the pronounced subjective discomfort (RPE/RPD), particularly during initial exposure, may negatively impact participant tolerance and training experience. Previous study suggests that the RPE and RPD during BFR exercise tend to decrease with repeated application of the technique, eventually approaching levels comparable to exercise without BFR [15]. Therefore, a more prudent LOP selection strategy may involve individualizing the applied pressure based on personal tolerance and adopting a progressive approach, starting from a lower percentage and gradually increasing it as tolerated.

No adverse events were reported during the study, which is consistent with existing published evidence regarding the safety of BFR training. Existing clinical studies on BFR exercise have been conducted across diverse populations, including hospitalized older adults, older adults with sarcopenia, and individuals with cardiovascular disease, with no serious BFR-related adverse events reported [33–35]. Even among patients with cardiovascular disease, BFR training has shown no serious adverse events directly attributed to the BFR intervention [36]. Therefore, under supervised conditions, BFR walking at 40%–80% LOP appears to be safe in screened sedentary college students.

### Implications for research

The findings of this study suggest several directions for future research on WT-BFR in sedentary college students. First, although the present study demonstrated that the acute hemodynamic response to WT-BFR was well tolerated, the chronic effects of repeated BFR exposure on cardiovascular function in sedentary college students remain unclear and warrant further investigation. Second, the potential long-term benefits of BFR-WT on musculoskeletal parameters—including muscle hypertrophy, strength gains, and functional capacity—have been documented across diverse populations, often surpassing those achieved through non-BFR training modalities [15,30,31]. However, as the present study does not directly address these chronic adaptations, further well-designed longitudinal studies are warranted to establish evidence-based recommendations. Third, studies should explore the physiological mechanisms of low-intensity WT-BFR, particularly its effects on vascular function, neuromuscular activation, and metabolic responses. Subsequent studies should adopt multidimensional health outcomes, including cardiovascular fitness, musculoskeletal health and metabolic function, to comprehensively evaluate the role of WT-BFR in promoting long-term health in sedentary college students.

### Implications for practice

The application of WT-BFR would be useful for its simplified, user-friendly devices that deliver low-intensity, efficient training protocols for sedentary college populations. The present findings indicate that low-intensity WT-BFR can be safely applied in sedentary college students, as no significant adverse hemodynamic responses were observed. However, perceptual responses warrant careful consideration, given that higher LOP levels (e.g., 80%) were associated with increased perceived exertion, discomfort and reduced step count. A more suitable strategy therefore involves selecting an appropriate LOP based on individual tolerance and progressively increasing the LOP%. Ultrasound-based individualized LOP% may enable precise BFR prescription and is recommended as a preferred approach for future research and practice. Furthermore, WT-BFR may confer time-efficient health benefits for sedentary college students, potentially achieving in a shorter duration the outcomes typically associated with guideline-recommended exercise volumes. Owing to its low-intensity nature, this modality is also well suited for populations with poor tolerance to moderate- intensity or high-intensity exercise, such as older adults with reduced physical function and patients with chronic conditions, highlighting its broader applicability [30,31]. Collectively, these advantages support WT-BFR as a practical adjunct to conventional low-intensity exercise programs, particularly for sedentary college students who may have limited tolerance for high-intensity exercise or exhibit low adherence to the traditional training programs required for optimal musculoskeletal adaptations.

### Strengths and potential limitations

This study presents several strengths. First, the randomized crossover design helped mitigate potential order bias. Second, the use of individualized LOP measurement based on Doppler ultrasound contributed to the precision of BFR application. Third, the inclusion of multiple hemodynamic and perceptual outcomes facilitated a comprehensive evaluation of the acute responses to WT-BFR.

There are several limitations in this study. Firstly, participants were recruited from a single institution, which may limit the generalizability of the findings. Future studies should expand the sampling scope and adopt multi-center recruitment strategies to improve sample heterogeneity and external validity. Second, this study employed an acute experimental design, and therefore cannot provide insights into the long-term effects or adaptations associated with WT-BFR training. Moreover, only three LOP levels (40%, 60%, and 80%) were examined, which may limit a comprehensive understanding of the dose–response relationship between LOP with physiological and perceptual outcomes. To address these limitations, future research should expand multi-center sampling, adopt longitudinal designs, and explore a broader range of LOP levels, thereby providing stronger evidence for safe and effective WT-BFR protocols in sedentary populations.

## Conclusions

The current investigation provides evidence supporting the viability and safety of WT-BFR as an alternative exercise modality for sedentary collegiate populations. The findings demonstrate that while perceived as physiologically demanding, WT-BFR elicit comparable cardiovascular stress profiles to non-BFR walking exercises of equivalent intensity, as evidenced by hemodynamic indicators. Analysis suggests an optimal BFR pressure range for this population, with 40% LOP recommended as the standard prescription while maintaining a maximum threshold of 60% LOP to ensure safety and tolerability. These findings represent an advancement in understanding the hemodynamic responses and physiological tolerability of WT-BFR, thereby contributing to the development of evidence-based strategies for enhancing physical fitness outcomes in sedentary college students. However, the long-term health effects of low-intensity WT-BFR among college students warrant further investigation.

## Supporting information

S1 Checklist CONSORT 2010 checklist. (DOCX)

S2 File Study protocol. (DOCX)
