# Supplementary material for: Effects of walking training with blood flow restriction on the hemodynamics and perceptual responses among sedentary college students: A randomized crossover trial
Source: PLoS One. 2026 Jul 13;21(7):e0352582. doi: 10.1371/journal.pone.0352582 (PMC13362102; doi:10.1371/journal.pone.0352582)
Supplement: S2 File — (DOCX) [file pone.0352582.s002.docx]

**Study Protocol**

**Title**

Effects of walking training with blood flow restriction on the hemodynamics and perceptual responses among sedentary college students: a crossover randomized trial

**Metadata**

**Funding**

This study was supported by the Innovation and Entrepreneurship Training Program of Soochow University (2024C029); Suzhou University School of Nursing (2024HLBK02); Suzhou Science and Technology Bureau (SKY2023124).

**Competing interests**

The authors declare that they have no competing interests.

**Data availability**

All code and result files used in this study are available. The authors agree to upload the original data when publishing.

**Abstract**

College students often present a sedentary lifestyle, which could be ameliorated by exercise such as walking training. Previous investigations have indicated that low-intensity walking training with blood flow restriction (WT-BFR) can deliver comparable health benefits with moderate-to-high intensity walking training without restriction in different populations. However, less is known regarding the potential benefit of WT-BFR in sedentary college students. This study aimed to examine the effects of WT-BFR at different limb occlusion pressure (LOP) on hemodynamic and perceptual responses in sedentary college students using a randomized crossover design. A total of 60 participants completed the 5-minute WT-BFR with varying LOPs (i.e., 0%, 40%, 60%, and 80%). Hemodynamic parameters (blood pressure and heart rate) were measured before, immediately after, and 5 minutes post-intervention. Meanwhile, perceptual responses (perceived exertion and discomfort) and step numbers were recorded post-intervention. For the hemodynamic parameters, only 60% LOP showed a larger increase in heart rate after training than 0% LOP (walk training without BFR) condition (*p*=0.016). Other hemodynamic parameters did not show statistical differences among all levels of LOP conditions. With the increase of LOP, perceived exertion and discomfort were increased significantly (*p*<0.05), and the step numbers were reduced (*p*<0.05). A five-minute session of WT-BFR at 40% to 80% of LOP did not elicit significant hemodynamic changes in college students. Based on participant perception, an LOP range of 40%–60% is recommended. The long-term health effects of low-intensity WT-BFR among college students warrant further investigation.

**Introduction**

College students exhibit a notably low level of physical activity and represent a typical sedentary population[1]. Studies have revealed that the average daily sedentary time among college students reaches 9.83 hours[2]. This sedentary lifestyle poses substantial health risks, including overweight/obesity[3], cancer[4], type 2 diabetes[5], cardiovascular disease mortality[5], and all-cause mortality[6]. Regular physical exercise has been demonstrated to substantially enhance both physiological and psychological well-being among college students, including improvements in cardiorespiratory fitness[7], sleep quality[8], and the alleviation of anxiety and depression[9]. However, the proportion of students engaging in regular exercise remains limited[1]. This phenomenon may be attributable to multiple exercise barriers, particularly academic-related factors such as time constraints and demanding coursework. In addition, subjective factors, including lack of personal exercise habits, limited interest, discomfort associated with high-intensity exercise, and insufficient sports skills, collectively contribute to reduced exercise participation[10].

The World Health Organization (WHO) recommended that adults aged 18-64 should complete at least 150 minutes of moderate-intensity aerobic activity or 75 minutes of vigorous-intensity aerobic activity weekly, supplemented by muscle-strengthening activities involving major muscle groups on two or more days per week[11]. However, the combination of limited time and various subjective factors collectively hinders college students from meeting the WHO's minimum threshold for health-promoting physical activity. As a fundamental form of physical activity, walking represents a substantial component of daily movement in this population. Nevertheless, it is typically performed at low intensity—a level that falls below the minimum recommended by the WHO.

Blood flow restriction (BFR) training has emerged as a scientifically reliable exercise modality, characterized by the application of specialized compression devices (e.g., pneumatic cuffs, elastic bands) to proximal limb segments, thereby reducing arterial inflow and blocking venous outflow to enhance training stimulus[12]. Walking training with BFR (WT-BFR) represents an innovative exercise paradigm that combines low-intensity dynamic training with controlled vascular occlusion. This time-efficient training method, characterized by its low intensity and short-duration parameters, has been demonstrated to be effective across diverse populations[13]. Typically, low-intensity exercise combined with BFR demonstrates superior effects compared to equivalent exercise without BFR, while producing outcomes comparable to high-intensity exercise without BFR. In overweight middle-aged males, WT-BFR has been shown to significantly improve systemic inflammatory markers, optimize lipid profiles, and modulate hematological indices[14]. For elderly populations, particularly those with knee osteoarthritis, WT-BFR not only exhibits excellent feasibility but also effectively enhances physical function[15, 16]. These findings underscore the significant application value of WT-BFR as a safe and effective exercise intervention for improving health outcomes in difference of populations.

Research indicates that the duration of a single BFR training session typically ranges from 5 to 20 minutes, with the BFR applied to both small and large muscle groups (e.g., upper and lower limbs, either unilaterally or bilaterall[17, 18]. The pressure for BFR is generally set between 40% and 80% of the limb occlusion pressure (LOP), where LOP is defined as the minimum pressure required to completely occlude blood flow in the limb. The exercise modalities primarily include aerobic exercise (e.g., cycling and walking) and resistance exercise (e.g., deep squat and knee extension)[19-21]. Existing studies suggest that higher BFR pressures may enhance cardiovascular responses but might also induce significant discomfort[22, 23]. Therefore, prior to implementing BFR training among sedentary college students, it is necessary to investigate the effects of different LOP levels on hemodynamic responses in this population.

WT-BFR may offer college students a low-intensity, time-efficient, and highly effective exercise modality. However, researches about WT-BFR on sedentary college students remains unreported. This study aims to investigate the effects of different intensities of BFR on hemodynamic and perception responses in sedentary college students and to evaluate the acceptability of various WT-BFR intensities in this population. The findings will help establish the safety profile of WT-BFR for sedentary college students and lay the groundwork for further research into the potential benefits of BFR exercise.

**Materials and Methods**

**Study goals and objectives**

The primary objective is to investigate the effects of different intensities of BFR on hemodynamic and perception responses in sedentary college students and to evaluate the acceptability of various WT-BFR intensities in this population. The secondary objective is to establish the safety profile of WT-BFR for sedentary college students and lay the groundwork for further research into the potential benefits of BFR exercise.

**Study design**

This study is a randomized crossover design. Participants were recruited at Soochow University by electronic poster from October 7th, 2024 to December 7th, 2024 (**Fig 1**).


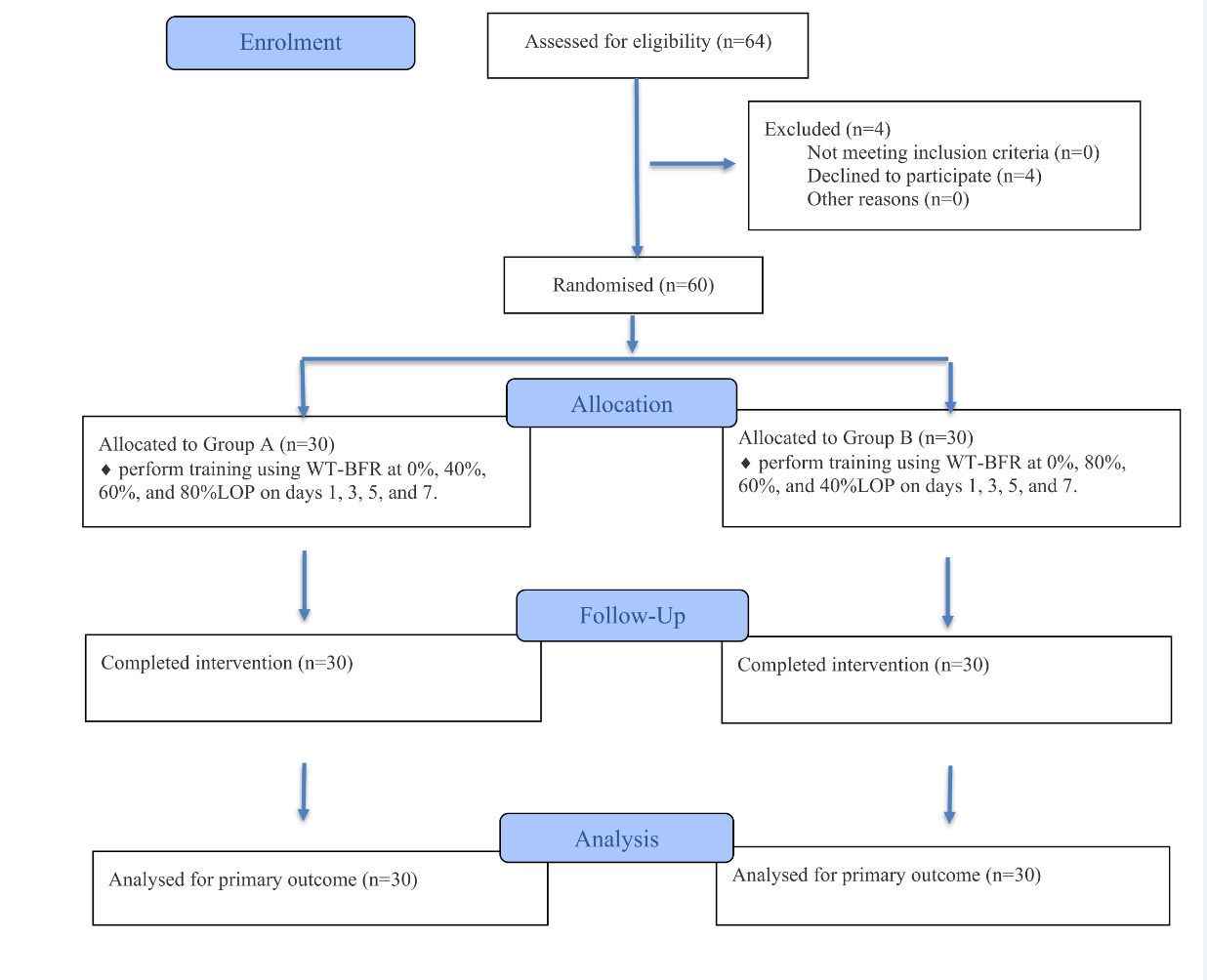
**Fig 1. CONSORT 2025 Flow Diagram.**

**Inclusion and exclusion criteria**

Eligibility criteria included: (ⅰ) college students; (ⅱ) low physical activity (<1500 MET-min/week assessed by International Physical Activity Questionnaire, IPAQ)[24]; (ⅲ) prolonged sedentary time (≥ 6 hours/day); (ⅳ) frequency exercise (<3 weekly sessions of <30 minutes duration); and (ⅴ) absence of exercise contraindications (e.g., severe cardiovascular diseases, respiratory diseases, musculoskeletal disorders, and metabolic diseases). Exclusion criteria were: (i) engaged in regular physical activity or exercise (>3 times/week of moderate to high-intensity exercise, each session lasting at least 20 minutes); (ⅱ) alcohol, nicotine, drug, and medications use; and (ⅲ) pregnant.

**Sample size calculation**

The sample size was calculated using a paired design-mean comparison algorithm $n=\frac{{(Z_{\alpha}+Z_{\beta})}^{2}*\sigma^{2}}{\delta^{2}}$[25]. According to published research, the standard deviation of systolic blood pressure (SBP) among college students was 15[26]. Based on the reference literature, a difference in SBP of less than 10mmHg between two different exercises is considered not significant. Hence, the mean difference between the two groups is set at 10mmHg. Type I error α was set at 0.05. Therefore, Z_0.05_=1.96. Type II error β was set at 0.1, Z_β_=1.28. The calculated sample size was 24 participants per group. Considering a 20% dropout rate, we planned to recruit 60 subjects.

**Expected duration**

The expected duration of the study is between October 7th, 2024 to December 7th, 2024. All data will be collected during this period.

**Methodology**

**Intervention protocol**

Participants will be randomly divided into two groups, with each group undergoing training under different conditions (**Fig 2**). Prior to the commencement of the formal trials, the limb occlusion pressure (LOP) of participants in both groups will be measured to confirm the pressure of BFR.


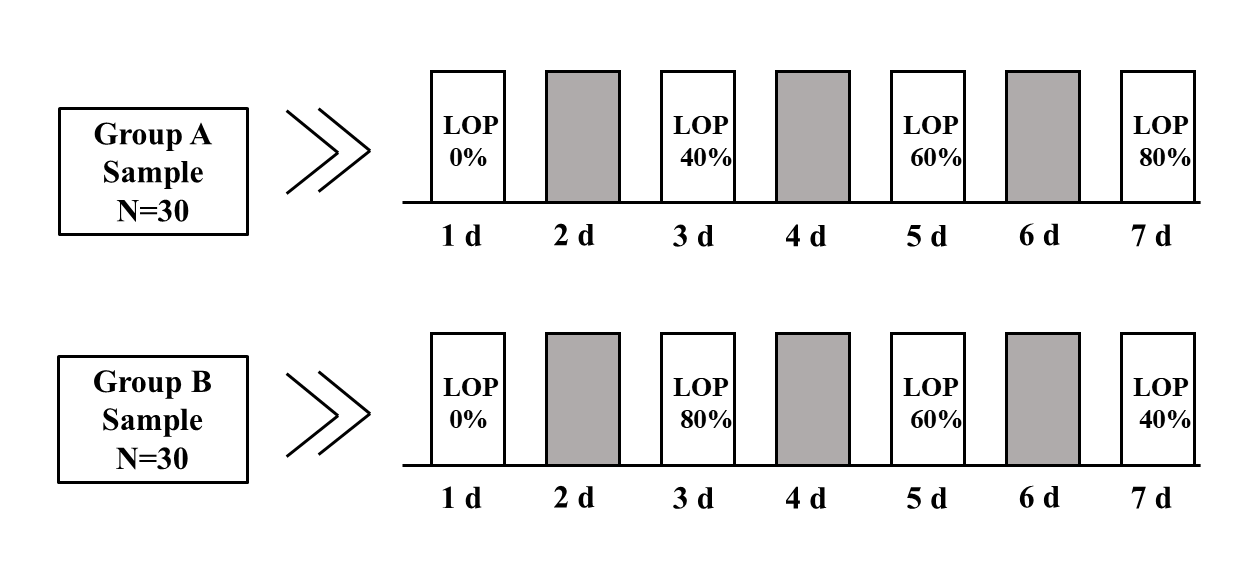


**Fig 2. Study Design**. The timing of exercise sessions is shown. LOP, limb occlusion pressure; shaded blocks, rest day.

Participants in Group A will perform training using WT-BFR at 0%, 40%, 60%, and 80%LOP on days 1, 3, 5, and 7 of the trial, respectively (**Fig 2**). Participants in Group B will perform training using WT-BFR at 0%, 80%, 60%, and 40%LOP on days 1, 3, 5, and 7 of the trial, respectively (**Fig 2**). Each training session is followed by a one-day interval before the next trial. The experiment will be conducted on a horizontal playground within Soochow University between 7:00 and 20:00 on each experimental day (days 1, 3, 5, 7) by two trained researchers.

The experimental procedure of each training session consists of three stages (**Fig 3**). Upon arrival at the test site, participants will rest for 5 minutes to ensure their bodies are in a calm state. After resting, the following outcomes will be measured, heart rate, SBP, diastolic blood pressure (DBP), mean arterial pressure (MAP), double product and pulse oxygen saturation (SpO_2_) (**Fig 3**). Following the data measurement, 5 minutes WT-BFR will commence. At the end of the intervention, hemodynamic data, rating of perceived exertion (RPE) [27], rating of perceived discomfort (RPD) [28] and step number will be immediately measured and recorded. After a further 5-minute rest, hemodynamic data will be recorded again (**Fig 3**).


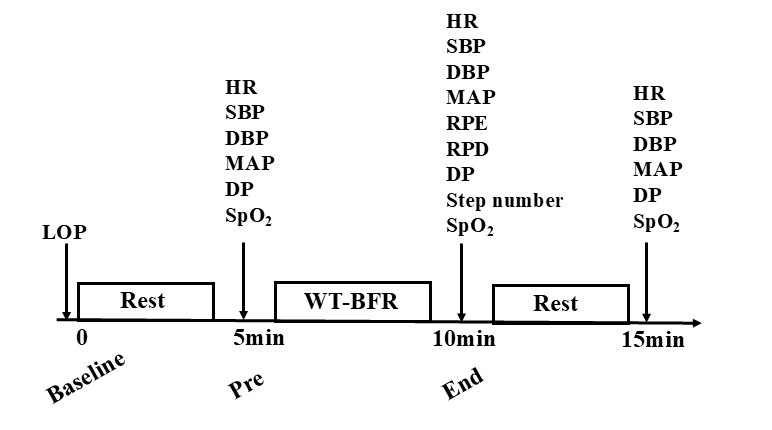
**Fig. 3. Timeline for each training session.** The timing of measures is indicated on the single session timeline. LOP, limb occlusion pressure; HR, heart rate; SBP, systolic blood pressure; DBP, diastolic blood pressure; MAP, mean arterial pressure; RPE, rating of perceived exertion; RPD, rating of perceived discomfort; SpO_2_, pulse oxygen saturation; WT-BFR, walking training with blood flow restriction; DP, double product.

**Blood flow restriction**

For conditions that required BFR, the restriction will be applied during each exercise bout only, using a uniform device: A&B Physiotherapy LTD, Theratools, China. The device is a 10 cm wide inflatable cuff with an external, removable, pressure-displaying, and manually inflatable deflator. Measurement of LOP will be completed before the first BFR exercise session. An inflatable cuff will be placed on the patient's upper thigh (near the groin) with an elasticity to accommodate two fingers in the uninflated state. A portable ultrasound monitor (Edan SD 3 Vascular Ultrasound Pocket Doppler, Edan USA) will be used to be placed on the ipsilateral intra-ankle anterior tibial artery course. When arterial pulsation is monitored, the inflatable cuff will be pressurized until the monitored arterial pulsation disappear, at which time the intracuff pressure is the LOP of the limb on that side. The LOP of the right and left lower limbs is measured and recorded separately. For the safety period, when the LOP is measured above 300 mmHg, the LOP is recorded as 300 mmHg[29].

**Outcomes measurements**

The primary outcome is SBP and second outcomes include hemodynamic indicators, perceived response, step number, and adverse events during training sessions.

Hemodynamic indicators including heart rate, blood pressure, and SpO_2_. For all sessions, hemodynamic measures will be taken immediately before, immediately after each exercise bout, and 5 minutes after rest. Hemodynamic measures include heart rate, SBP, DBP, MAP, double product, and SpO_2_. Heart rate, SBP, and DBP will be measured using a smart blood pressure monitor (Yuwell, YE660AR, Danyang China). Double product is calculated using the formula DP=SBP × HR, MAP is calculated using the formula MAP = (SBP + 2 × DBP) / 3[30], and SpO_2_ will be measured using a medical pulse oximeter (Yuwell, YX306, Danyang China).

Perceptual response including RPE, RPD. Immediately after each exercise bout, participants will be asked to provide RPE on a Borg scale ranging from 6 (no exertion) to 20 (maximal exertion) and RPD using a modified Borg scale ranging from 0 (no discomfort) to 10 (maximal discomfort). RPD using a modified Borg scale ranging from 0 (no discomfort) to 10 (maximal discomfort).

Step number will be recorded during each training sessions.

**Randomization**

An independent research team member numbered the subjects in the order they are included, used IBM.SPSS23.0 software to generate random numbers corresponding to each subject, arrange the random numbers from smallest to largest, and divide the subjects of different genders into group A (50%) and Group B (50%) according to a 1:1 ratio.

Details of group allocation will be kept confidential on cards placed inside a sequentially numbered series of sealed opaque envelopes. Another independent research assistant will be responsible for maintaining these sealed envelopes, contacting participants, and assigning them to their designated groups.

**Blinding**

This study is a single-blind study where the assessors are blinded. Due to the characteristics of this study, complete blinding of the participants and the implementers is not feasible. Some open trial designs may cause bias in the participants' compliance and participation, as well as in the implementation, thereby affecting the research results. Therefore, this study will take some measures to reduce the above biases. To reduce the bias of expected effects, the hypotheses of the study and the specific operational methods will not be informed to the participants in advance. The information provided in the participant's informed consent form is all without a clear effect indication. For the measurement of outcome indicators before and after exercise (cardiovascular indicators, such as blood pressure and heart rate), standard equipment and unified standard operating procedures will be used to reduce measurement errors. For the assessment of primary and secondary outcome indicators, the assessors will be blinded (that is, the assessors do not know the participant's group at any stage, and the participants are also informed not to inform the assessors of their group situation in any way).

**Data management plans**

All research data generated in this study will be managed in accordance with the FAIR principles (Findable, Accessible, Interoperable, and Reusable).

**Data Collection**

Data will be collected by trained researchers during the intervention phase (October–December 2024). All data will be recorded using standardized forms and validated instruments. The data entry will be checked by two other researchers to ensure its accuracy.

**Storage and Backup**

All electronic data will be stored on a password-protected secure server at Soochow University, accessible only to authorized study personnel. Regular backups will be performed weekly to an external encrypted hard drive and a cloud-based institutional storage system. At least three copies of the dataset will be maintained throughout the study.

**Selection and Preservation**

The final curated dataset will be retained for a minimum of 10 years after study completion. Data will be preserved in non-proprietary formats (e.g., TXT, PDF) to ensure long-term accessibility. Relevant documentation, including the study protocol, statistical analysis plan, and informed consent templates, will also be archived.

**Ethics and Legal Compliance**

This study was approved by the Soochow University Ethics Committee (No. SUDA20241006H03) and registered with the China Clinical Trial Registry (ChiCTR2500097728). All participants provided written informed consent. Data management practices comply with the Personal Information Protection Law of China and international standards for clinical trial data integrity. No personally identifiable information will be shared publicly.

**Responsibilities**

The Principal Investigator (Huagang Hu) is responsible for overall data management. Yuke Zhu will oversee daily data collection, validation, and archiving. Access to raw data is restricted to the core research team. Any data sharing requests from external researchers will be reviewed by the PI and must comply with ethical and legal requirements.

**Safety considerations**

Any physical discomfort such as chest pain, chest tightness, dyspnea, pain in the lower limbs, symptomatic high or low blood pressure, and mental discomfort will be recorded and reported back to the research team and the doctor for timely management. At the end of the BFR exercise, the researcher will check the condition of the skin at and below the cuff coverage area, such as bruising or edema.

**Data management and statistical analysis**

The database was established using Epidata 3.1. The statistical analyses were performed using SPSS 25.0 (IBM, Chicago, IL). Normally distributed measurement data are described using mean ± standard deviation (SD), while non-normally distributed data are described using median and interquartile range [M (P_25_, P_75_)]. Categorical data are described using frequency (percentage). One-way ANOVA was used to compare the hemodynamic indicators, RPE, RPD, and step number among different percentage LOP conditions at each time point. Generalized estimating equation (GEE) model was used to analysis the effects of different levels of LOP on hemodynamics, RPE, and RPD in sedentary college students. Two-side statistically significant level was set at 0.05.

**Ethics**

The trial protocol was approved by the Soochow University Ethics Committee (Approval No.: SUDA20241006H03) on October 6th, 2024. This trial has been registered on the China Clinical Trial Registry (Registration number: ChiCTR2500097728 25/02/2025). Before the study, written informed consent will be obtained from each participant.

**Study timeline**

**2024.09-2024.10**: Obtain the approval of the ethics committee, print the relevant scales and train relevant researchers.

**2024.10-2025.12**: Recruit participants, conduct the intervention and collect datas.

**2025.12-2025.03**: Import data and analyze data.

**2025.04-2025.07**: Write a thesis paper.

**Discussion**

**Limitations**

There are several limitations in this study. Firstly, the recruitment of participants was limited to a single institution, which may result in insufficient sample representativeness. This limitation, to some extent, restricts the generalizability of the research findings to the broader population of sedentary college students. It is recommended that subsequent studies expand the sampling scope and adopt a multi-center recruitment strategy to enhance the heterogeneity and representativeness of the sample, thereby strengthening the universality of the research conclusions. Secondly, in terms of research design, the inability to implement blinding due to objective constraints may lead to experimenter effects and participant bias, potentially compromising the objectivity of the research outcomes. Additionally, critical methodological parameters—including reliability analyses, measurement error metrics (standard error of the measurement), and minimal detectable change values—were not reported. Given these limitations, it is suggested that future research should focus on optimizing experimental designs, improving the implementation of blinding, increasing critical methodological parameters, and employing stratified sampling methods. Such improvements would facilitate a more in-depth investigation into the dose-response relationship, thereby providing more reliable evidence-based support for the development of scientifically sound and efficient low-intensity BFR exercise protocols for sedentary college students and even broader sedentary populations.

**Dissemination Plans**

Publish peer review paper.

**Protocol Amendments and Termination**

If unforeseen intervention-related risks, adverse reactions, or feasibility issues (e.g., recruitment difficulties, high withdrawal rates, or data collection obstacles) arise during the study, necessitating changes to data collection procedures, the informed consent form, sample size, study sites, or duration, such substantive amendments must be submitted to the Soochow University Ethics Committee and the Chinese Clinical Trial Registry for formal approval prior to implementation. All amendments will be documented in detail, and the protocol version will be updated accordingly. If an amendment directly affects participants' rights and interests, informed consent will be re-obtained.

The study may be terminated prematurely under the following circumstances: (1) identification of major safety concerns; (2) definitive evidence of efficacy or futility; (3) insurmountable feasibility issues. Upon termination, all study activities will cease immediately, participants will be notified, and a final report will be submitted to both the Ethics Committee and the Registry. All collected data will be securely preserved and appropriately analyzed.

**Authors’ contributions**

**Conceptualization:** Yuke Zhu, Huagang Hu.

**Data collection:** Yuke Zhu, Ying Wang, Siyu Yu, Dejiang Sun, Xiuyi Ji, Wenbin Yuan, Min Ma.

**Data curation:** Yuke Zhu, Ying Wang, Huagang Hu.

**Formal analysis:** Yuke Zhu, Ying Wang, Huagang Hu.

**Methodology:** Yuke Zhu, Ying Wang, Huagang Hu.

**Software:** Yuke Zhu, Huagang Hu.

**Project administration:** Yuke Zhu, Huagang Hu.

**Writing-original draft:** Yuke Zhu, Ying Wang, Huagang Hu.

**Writing-review and editing:** Yuke Zhu, Huagang Hu.

**Supervision:** Huagang Hu.

**Acknowledgements**

The authors thank all participants and assistance from medical writers, proof-readers and editors. This study was supported by the Project of Suzhou Key Laboratory of Geriatric Intelligent nursing and health preservation.

**Supporting Information**

NA.

**References**

1. Carballo-Fazanes A, Rico-Díaz J, Barcala-Furelos R, Rey E, Rodríguez-Fernández JE, Varela-Casal C, Abelairas-Gómez C. Physical Activity Habits and Determinants, Sedentary Behaviour and Lifestyle in University Students. Int J Environ Res Public Health. 2020;17(9).

2. Maher JP, Doerksen SE, Elavsky S, Conroy DE. Daily satisfaction with life is regulated by both physical activity and sedentary behavior. J Sport Exerc Psychol. 2014;36(2):166-78.

3. Tremblay MS, Willms JD. Is the Canadian childhood obesity epidemic related to physical inactivity? Int J Obes Relat Metab Disord. 2003;27(9):1100-5.

4. Thorp AA, Owen N, Neuhaus M, Dunstan DW. Sedentary behaviors and subsequent health outcomes in adults a systematic review of longitudinal studies, 1996-2011. Am J Prev Med. 2011;41(2):207-15.

5. Wilmot EG, Edwardson CL, Achana FA, Davies MJ, Gorely T, Gray LJ, et al. Sedentary time in adults and the association with diabetes, cardiovascular disease and death: systematic review and meta-analysis. Diabetologia. 2012;55(11):2895-905.

6. Grøntved A, Hu FB. Television viewing and risk of type 2 diabetes, cardiovascular disease, and all-cause mortality: a meta-analysis. Jama. 2011;305(23):2448-55.

7. Chiu YH, Tsai SC, Lin CS, Wang LY, Huang KC. Effects of a 12-week walking intervention on circulating lipid profiles and adipokines in normal weight and abdominal obese female college students. J Exerc Sci Fit. 2023;21(3):253-9.

8. Li L. Effects of Aerobic Exercise on Sleep Quality and Mental Health of College Students. Occup Ther Int. 2022;2022:8366857.

9. Huang K, Beckman EM, Ng N, Dingle GA, Han R, James K, et al. Effectiveness of physical activity interventions on undergraduate students' mental health: systematic review and meta-analysis. Health Promot Int. 2024;39(3).

10. Ferreira Silva RM, Mendonça CR, Azevedo VD, Raoof Memon A, Noll P, Noll M. Barriers to high school and university students' physical activity: A systematic review. PLoS One. 2022;17(4):e0265913.

11. WHO Guidelines Approved by the Guidelines Review Committee. WHO Guidelines on Physical Activity and Sedentary Behaviour. Geneva: World Health Organization

© World Health Organization 2020.; 2020.

12. Kim J, Lang JA, Pilania N, Franke WD. Effects of blood flow restricted exercise training on muscular strength and blood flow in older adults. Exp Gerontol. 2017;99:127-32.

13. LaPrade RF, Monson JK, Schoenecker J. Editorial Commentary: Blood Flow Restriction Therapy Continues to Prove Effective. Arthroscopy. 2021;37(9):2870-2.

14. Razi O, Mohammadi M, Zamani N, Hackney AC, Tourny C, Zouita S, et al. Walking exercise and lower-body blood flow restriction: Effects on systemic inflammation, lipid profiles and hematological indices in overweight middle-aged males. Res Sports Med. 2022;30(1):41-9.

15. Clarkson MJ, Conway L, Warmington SA. Blood flow restriction walking and physical function in older adults: A randomized control trial. J Sci Med Sport. 2017;20(12):1041-6.

16. Petersson N, Langgård Jørgensen S, Kjeldsen T, Mechlenburg I, Aagaard P. Blood Flow Restricted Walking in Elderly Individuals with Knee Osteoarthritis: A Feasibility Study. J Rehabil Med. 2022;54:jrm00282.

17. Manini TM, Clark BC. Blood flow restricted exercise and skeletal muscle health. Exerc Sport Sci Rev. 2009;37(2):78-85.

18. Larkin KA, Macneil RG, Dirain M, Sandesara B, Manini TM, Buford TW. Blood flow restriction enhances post-resistance exercise angiogenic gene expression. Med Sci Sports Exerc. 2012;44(11):2077-83.

19. Patterson SD, Hughes L, Warmington S, Burr J, Scott BR, Owens J, et al. Blood Flow Restriction Exercise: Considerations of Methodology, Application, and Safety. Front Physiol. 2019;10:533.

20. Lixandrão ME, Ugrinowitsch C, Berton R, Vechin FC, Conceição MS, Damas F, et al. Magnitude of Muscle Strength and Mass Adaptations Between High-Load Resistance Training Versus Low-Load Resistance Training Associated with Blood-Flow Restriction: A Systematic Review and Meta-Analysis. Sports Med. 2018;48(2):361-78.

21. Centner C, Wiegel P, Gollhofer A, König D. Effects of Blood Flow Restriction Training on Muscular Strength and Hypertrophy in Older Individuals: A Systematic Review and Meta-Analysis. Sports Med. 2019;49(1):95-108.

22. Jessee MB, Dankel SJ, Buckner SL, Mouser JG, Mattocks KT, Loenneke JP. The Cardiovascular and Perceptual Response to Very Low Load Blood Flow Restricted Exercise. Int J Sports Med. 2017;38(8):597-603.

23. Mattocks KT, Jessee MB, Counts BR, Buckner SL, Grant Mouser J, Dankel SJ, et al. The effects of upper body exercise across different levels of blood flow restriction on arterial occlusion pressure and perceptual responses. Physiol Behav. 2017;171:181-6.

24. Hallal PC, Victora CG. Reliability and validity of the International Physical Activity Questionnaire (IPAQ). Med Sci Sports Exerc. 2004;36(3):556.

25. Lachin JM. Introduction to sample size determination and power analysis for clinical trials. Control Clin Trials. 1981;2(2):93-113.

26. Rui Kang et al. Analysis of factors affecting and correlating anxiety and blood pressure among college students at school. In: Kang R, Wang X, Shan H, He X, Ma Y, Duan Y, editors. Journal of Inner Mongolia Medical University2016. p. 45-9+53.

27. Borg GA. Perceived exertion. Exerc Sport Sci Rev. 1974;2:131-53.

28. Borg GA. Psychophysical bases of perceived exertion. Med Sci Sports Exerc. 1982;14(5):377-81.

29. Cook SB, Clark BC, Ploutz-Snyder LL. Effects of exercise load and blood-flow restriction on skeletal muscle function. Med Sci Sports Exerc. 2007;39(10):1708-13.

30. Shapiro DS, Loiacono LA. Mean arterial pressure: therapeutic goals and pharmacologic support. Crit Care Clin. 2010;26(2):285-93, table of contents.
